# Framework for the evaluation of new tests for tuberculosis infection

**DOI:** 10.1183/13993003.04078-2020

**Published:** 2021-08-19

**Authors:** Yohhei Hamada, Saskia den Boon, Daniela Maria Cirillo, Adam Penn-Nicholson, Morten Ruhwald, Dick Menzies, Olivia Oxlade, Dennis Falzon, Avinash Kanchar, Alexei Korobitsyn, Matteo Zignol, Alberto Matteelli, Tereza Kasaeva

**Affiliations:** 1 Research Institute of Tuberculosis, Japan Anti-Tuberculosis Association, Tokyo, Japan; 2 University College London, London, UK; 3 Global TB Programme, World Health Organization, Geneva, Switzerland; 4 IRCCS San Raffaele Scientific Institute, Milan, Italy; 5 Foundation for Innovative New Diagnostics (FIND), Geneva, Switzerland; 6 McGill International TB Centre, Montreal, QC, Canada; 7 Collaborating Centre for TB/HIV Co-infection and TB Elimination, Dept of Infectious and Tropical Diseases, University of Brescia, Brescia, Italy

## Abstract

The scale-up of tuberculosis (TB) preventive treatment (TPT) must be accelerated to achieve the targets set by the United Nations High-level Meeting on TB and the End TB Strategy. The scale-up of effective TPT is hampered by concerns about operational challenges to implement the existing tests for TB infection. New simpler tests could facilitate the scale-up of testing for TB infection. We present a framework for evaluation of new immunodiagnostic tests for the detection of TB infection, with an aim to facilitate their standardised evaluation and accelerate adoption into global and national policies and subsequent scale-up. The framework describes the principles to be considered when evaluating new tests for TB infection and provides guidance to manufacturers, researchers, regulators and other users on study designs, populations, reference standards, sample size calculation and data analysis and it is also aligned with the Global Strategy for TB Research and Innovation adopted by the World Health Assembly in 2020. In addition, we briefly describe technical issues that should be considered when evaluating new tests, including the safety for skin tests, costs incurred by patients and the health system, and operational characteristics.

## Abstract 2

**The evaluation of new, improved tests for tuberculosis infection should be expedited using standard study protocols to accelerate adoption into policy and subsequent scale-up. A framework for such evaluation is described.** https://bit.ly/38ChJbe

## Introduction

Treatment of tuberculosis (TB) infection, also known as tuberculosis preventive treatment (TPT), is a critical component needed to achieve the ambitious targets of the End TB Strategy 2016–2035 [1]. Management of TB infection is also critical to pursue TB elimination [2]. Without the prospect that a new, efficacious and safe TB vaccine can be developed and scaled-up worldwide in the foreseeable future, continued measures to expand the provision of effective TPT remain of critical importance. Furthermore, at the first United Nations High-level Meeting on TB in 2018, member states committed to provide TPT to ⩾30 million people in 2018–2022: 6 million people living with HIV (PLHIV), 4 million children aged <5 years who are household contacts of people with TB and 20 million other household contacts [3]. Progress in expanding TPT coverage among contacts of TB patients has been very limited up to now, with approximately half a million contacts in 2019 [4]. There is no gold standard method for diagnosing TB infection [5]. The World Health Organization (WHO) currently recommends a tuberculin skin test (TST) or an interferon-γ release assay (IGRA) to test for TB infection [6]. The tests are helpful to identify people at higher risk of developing TB disease and who could benefit from TPT, because in most published studies such risk is higher in people who test positive for TB infection than in those who test negative [7]. However, current tests for TB infection have limited value in predicting the risk of progression from infection to active TB disease [8].

The development and evaluation of tests characterised by higher prediction capacity (called tests of progression) is a high priority for research. In 2018, the WHO published a guidance document on the characteristics of such tests [9]. According to the target product profile defined by the WHO, optimal sensitivity and specificity of such tests for predicting development of TB disease are ⩾90% [9, 10], much higher than those of the currently available TST or IGRA [11]. Until better tests become available for use under field conditions, existing tests for TB infection, including TST and IGRA, remain the standard tests of choice.

**FIGURE 1:**
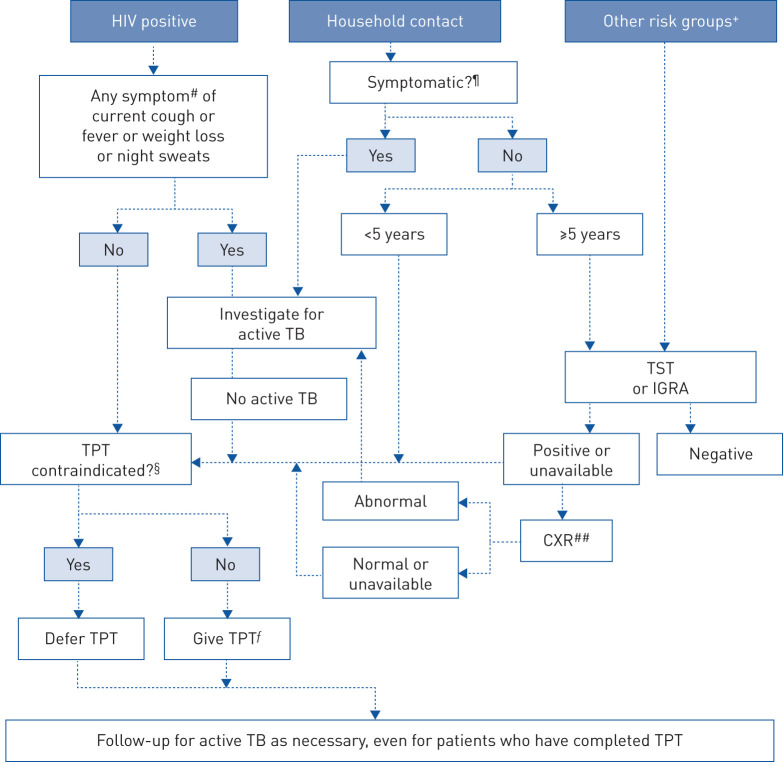
Algorithm for testing and treating tuberculosis (TB) infection in different groups considered to be at risk. ^#^: if aged <10 years, any one of current cough or fever or history of contact with a person diagnosed with TB or reported weight loss or confirmed weight loss >5% since last visit or growth curve flattening or weight for age <−2 z-scores. Asymptomatic infants with HIV aged <1 year are only treated for TB infection if they are household contacts of TB. Tuberculin skin test (TST) or an interferon-γ release assay (IGRA) may identify people living with HIV (PLHIV) who will benefit most from preventive treatment. Chest radiography (CXR) may be used in PLHIV on antiretroviral therapy, before starting treatment of TB infection. ^¶^: any one of cough or fever or night sweats or haemoptysis or weight loss or chest pain or shortness of breath or fatigue. In children aged <5 years, they should also be free of anorexia, failure to thrive, not eating well, decreased activity or playfulness to be considered asymptomatic. ^+^: including silicosis, dialysis, anti-tumour necrosis factor agent treatment, preparation for transplantation or other risks in national guidelines. ^§^: including acute or chronic hepatitis, peripheral neuropathy (if isoniazid is used) or regular and heavy alcohol consumption. Pregnancy or a previous history of TB are not contraindications. ^ƒ^: regimen chosen based on considerations of age, strain (drug susceptible or otherwise), risk of toxicity, availability and preferences. ^##^: CXR may have been carried out earlier on as part of intensified case finding. TPT: TB preventive treatment. Reproduced and modified from [6] with permission.

Tests for TB infection are not required before starting TPT in people from high-priority groups such as PLHIV and household contacts aged <5 years in high TB burden countries (figure 1) [6]. For people from other at-risk populations, tests for TB infection are recommended to identify those who would benefit most from treatment and to avoid unnecessary treatment, which carries a risk of adverse events (figure 1). However, implementation of tests for TB infection is fraught with difficulties, including short supply of quality-assured purified protein derivative, the need for training to perform and read TST, inadequate laboratory set-up to undertake IGRA testing in decentralised settings and high costs (IGRA). This calls for new tests with better operational characteristics.

New versions of TST and IGRA are already on the market or in the pipeline, all using ESAT6 and CFP10 antigens. Diaskintest (Generium, Moscow, Russia) [12] and ESAT6-CFP10 (Anhui Zhifei Longcom Biopharmaceutical Co. Ltd, Anhui, China) [13], both of which are IGRA-like skin tests for TB infection, are commercially available, and a new IGRA-like skin test, C-Tb (Serum Institute of India, Pune, India) [14, 15], has recently been developed. QIAGEN (Venlo, the Netherlands), the manufacturer of the IGRA test QuantiFERON-TB Gold Plus, and SD Biosensor (Suwon-Si, Republic of Korea) have both developed simplified versions of IGRA that can operate with less sophisticated laboratory support (M. Ruhwald; “Future tests for TB infection”, presented at the 51st Union World Conference on Lung Health; 20–24 October 2020).

Evaluation of tests for TB infection is not straightforward, due to the lack of a reference standard. Here we present a framework for evaluation of new immunodiagnostic tests for the detection of TB infection, with an aim to facilitate their standardised evaluation and accelerate adoption into global and national policy and subsequent scale-up. The document is primarily intended to guide the work of manufacturers of diagnostics, researchers, funders, regulators, TB programme coordinators, civil society and other stakeholders. The framework was developed by a WHO-convened technical expert group with inputs from reviewers with expertise in this field [16].

The focus of this framework is on the evaluation of diagnostic performance of tests for TB infection. Additionally, we briefly outline technical issues that should be considered when evaluating new tests for TB infection, evaluation of safety for skin tests, costs incurred by patients and the health system and operational characteristics.

## Framework for evaluation of new tests for TB infection

**FIGURE 2:**
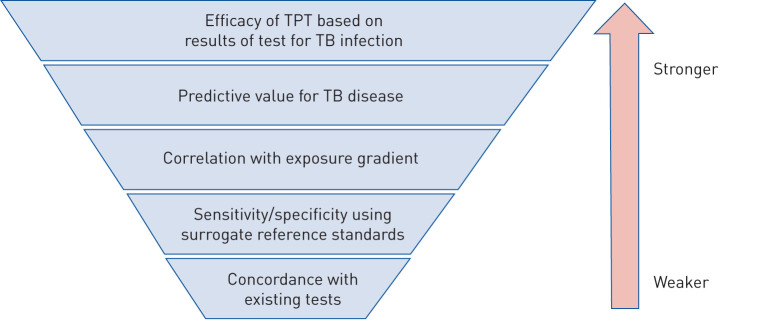
Hierarchy of reference standards used to assess the evidence base for tests for tuberculosis (TB) infection. TPT: TB preventive treatment. Reproduced and modified from [16] with permission.

The lack of an adequate gold standard complicates the estimation of sensitivity and specificity, the standard approach to assess the performance of new diagnostic tests. Surrogate reference standards are therefore proposed. A hierarchy of reference standards was developed when the WHO reviewed evidence on the use of IGRA (figure 2) [11].

Reference standards at higher levels of hierarchy should provide stronger evidence for a test's accuracy to identify people who would benefit most from TPT. New tests for TB infection currently in the pipeline are based on similar concepts to TST and IGRA; that is, eliciting an immune response to *Mycobacterium tuberculosis*-specific antigens either *in vivo* (size of skin induration) or *in vitro* (magnitude of cytokine release) [17]. Therefore, they are not expected to offer significant advantage in predicting risk of progression to TB disease. In general, the predictive performance of any new test for TB infection should not be inferior to current technology. Given the challenges to measure predictive value (see later), studies comparing sensitivity, specificity and concordance between new and current tests may be the best feasible option. In this case, a new test for TB infection could demonstrate noninferior sensitivity and specificity based on a pre-specified margin or concordance compared with at least one of the currently available tests endorsed by the WHO as rule-in tests for TPT. Because there is mounting evidence that IGRA has higher specificity and possibly higher sensitivity than TST [18, 19], IGRA should be preferred as a comparator in new trials [14, 20]. Any new tests intended to achieve significant improvement in predictive performance should follow the evaluation framework for tests for predicting progression to TB disease [9, 10].

## Study design and population

### Study design 1: predictive performance

**FIGURE 3:**
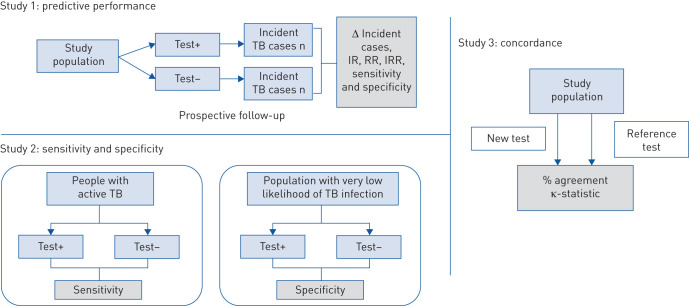
Study designs for the evaluation of tests for tuberculosis (TB) infection. Studies 1 and 2 include a single test in the figure for simplicity, but a new test should be compared with a reference test. Study design 1 is based on Kik *et al.* [9]; see therein for further details. IR: incidence rate; RR: risk ratio; IRR: incidence rate ratio.

A prospective longitudinal study measuring predictive value is the most appropriate method to compare performance of tests for TB infection, and could thus inform future policy on the use of new tests (figure 3). The WHO recommendation to use either TST or IGRA to test for TB infection is based on a review of this type of study [6, 21]. Using this study design, people with positive or negative tests for TB infection are screened for TB disease; those who are free of TB disease are then followed for ⩾12 months for development of TB disease. Follow-up of ⩾12 months is recommended to ensure a sufficient length of time for progression to occur. The major ethical issue with this design is that people at risk of TB with a positive test for TB infection should be started on TPT, which interferes with the primary end-points such as TB incidence or mortality. Although TB incidence can be measured in people who test positive for TB infection but who do not take treatment, this will reduce the study power and may also introduce bias given that people opting out may differ in characteristics associated with end-points from those who do take treatment. Moreover, the study design may preclude masking of the study subjects (*e.g.* comparing a new TST to IGRA). In addition, people who do not follow the recommendations from the provider are more likely to discontinue, which may seriously jeopardise the integrity of the cohort design. However, a relative (not absolute) estimate of test predictive performance may still be derived for people opting out of TPT, if two or more tests are evaluated simultaneously in the same cohort. Detailed guidance on how to conduct such studies is available in the framework for evaluation of tests for progression [9, 10].

### Study design 2: sensitivity and specificity using clinical reference standards

Sensitivity can be assessed using culture-confirmed TB as a reference standard. This is important to reduce bias in the estimated accuracy. It should be encouraged even for paediatric TB and forms of extrapulmonary TB that tend to be diagnosed clinically. A diagnosis based only on clinical or radiographic criteria should only be exceptional. In this case, it is important not to include tests for TB infection in the diagnostic criteria as this will spuriously overestimate the sensitivity of the test for infection.

Specificity depends on the antigens used to induce the immune response. By using overlapping peptides from antigens that are highly *M. tuberculosis*-specific, such as ESAT6 and CFP10, current IGRA tests are not affected by bacille Calmette–Guérin (BCG) vaccination, unlike TST, and their specificity is high at 97–99% based on studies conducted in low-risk populations who are unlikely to have been infected with TB. However, expression of ESAT6 and CFP10 by a group of nontuberculous mycobacteria species, including *M. marinum* and *M. kansasii*, is well known to lead to false-positive test results in people infected with these species. For the estimation of specificity, the population is one with very low likelihood of prior exposure to *M. tuberculosis*. It is important to evaluate the impact of cross-reactions by conducting subgroup analyses by BCG vaccination status and by likelihood of exposure to nontuberculous mycobacteria; for example, based on geographical and demographic information [22]. For assessment of tests for TB infection based on TB-specific antigens not found in the BCG vaccine itself (such as ESAT6 or CFP10), vaccination status of participants does not need to be taken into consideration.

The tests should be performed at the same time as the clinical evaluation as a delay between them can introduce bias [22]. This can be part of a cohort study, but longitudinal follow-up is not essential. In addition, it is important to conduct the tests in different TB epidemiological settings, although specificity cannot be measured in settings with high prevalence of TB infection. As discussed earlier, estimation of sensitivity and specificity entails two different cohorts, people with active TB and individuals with very low likelihood of TB infection. Participants in each cohort should be selected randomly or consecutively when enrolled. It is particularly important to avoid selecting the study population from a larger group of potentially eligible people on the basis of clinical or disease characteristics that might affect test performance (although this is acceptable in early stages of the evaluation of new tests).

The tests should be performed by well-trained people experienced in the procedures for both the reference test and the new test. People performing and evaluating tests should be blinded to the results of the other tests. Methods for supervision, monitoring and quality control should be adequate and clearly described. These points also apply to studies of concordance.

### Study design 3: concordance of tests

This design is essentially a study of agreement between new and reference tests and is the lowest level of evidence for assessment of diagnostic tests. It should be noted that concordance may be low when new tests are thought to have superior predictive performance or sensitivity and specificity. Therefore, this design is appropriate when the new test offers operational advantages over existing tests, but no gain in diagnostic performance is expected.

Study participants should be representative of the general population. For example, it is important to include very young or elderly people, pregnant women, people with more severe disease or people with serious comorbidities (*e.g.* HIV, diabetes, renal failure, malnutrition), as these may affect test performance. Studying agreement in different TB epidemiological settings and expected burdens of nontuberculous mycobacteria is encouraged.

When two tests are administered, they should be done at the same time, ideally on the same sample (if based on blood or urine). If analysing skin tests, the reference and index tests should be administered in each arm at the same time. Collection of blood or urine specimens for *in vitro* tests for TB infection should be done at the same time or before administering a TST to avoid a boosting effect [23].

## Sample size calculation

### Principles of sample size determination: superiority *versus* noninferiority

Superiority designs and related sample size calculations are appropriate if the reference test has suboptimal performance. For example, the sensitivity of current tests for TB infection is judged to be suboptimal. Sensitivity is particularly suboptimal in young children, PLHIV and otherwise immunocompromised people, who are at increased risk of disease and therefore a priority for testing for TB infection. Therefore, a new test would be of great interest if it had superior sensitivity, particularly in these high-risk populations.

If the reference test has excellent specificity, such as the 97–99% specificity of current IGRAs, it would require a large sample size to demonstrate superiority (table 1) [24]. However, a new test could have specificity that is noninferior to an existing test, and will be preferred because of other advantages. If a noninferiority design is selected, these other important advantages, such as lower cost to the patient or health system, enhanced feasibility, or point-of-care availability, must be pre-specified, and measured carefully. It is also important to note that noninferiority designs should be reserved to test performance when the standard or reference test has very good performance, such as the example of the specificity of the IGRA tests. This is necessary to avoid a risk for the progressive deterioration of test performance over time if newly emergent technologies are successively compared to the previous generation of test using noninferiority criteria [25].

**TABLE 1:** Example of changes in different key assumptions on sample size requirements to demonstrate superiority/noninferiority of tests for sensitivity and specificity

	**Reference**	**New test**	**Difference**	**Sample size required** ^#^ **(80% power) n**
**Sensitivity**	95	98	3	331
	95	92	3	478
	95	90	5	185
	95	87	8	79
	90	93	3	716
	90	87	3	843
	90	85	5	316
	90	82	8	130
**Specificity**	98	99	1	1283
	98	95	3	233
	98	93	5	96
	98	90	8	44
	95	98	3	331
	95	92	3	478
	95	90	5	185
	95	87	8	79

Data are presented as %, unless otherwise stated. From www.stat.ubc.ca/~rollin/stats/ssize/b1.html sample size calculator website. ^#^: total number of participants, since both tests are performed in the same persons.

### General determinants of sample size

When designing a study, it is particularly important to pre-specify the effect size, or difference expected, for both superiority and noninferiority designs. In general, the larger the effect size, the smaller the sample size. It is important to recognise that larger effect sizes may not be realistic and may lead to erroneous conclusions. In a study with too few participants, a new test that has superior performance, but by a small difference from the reference test, may fail to show superiority due to a wide confidence interval crossing the null value. The estimates of performance of the reference test used for sample size calculation should be based on recently conducted high-quality systematic reviews to ensure their accuracy.

## Specific sample size calculations and study analysis

### Predictive performance

The new test would ideally have good sensitivity and reasonable specificity. However, new tests for TB infection are not expected to improve predictive value substantially, and most gains are expected to be seen in operational aspects. Hence, demonstrating noninferiority in terms of predictive performance would be acceptable.

Sample size calculations have to account for the likelihood of future TB disease, as this determines the number of events, the sensitivity of current tests in predicting these events, and differences in sensitivity of the new test and follow-up time. While longer periods of follow-up reduce the required sample size, it may result in greater losses to follow-up and lead to higher risk of reinfection in high-transmission settings, which could complicate interpretation of the initial test. Hence, longer periods of follow-up are not encouraged, at least for the primary analysis and sample size calculations.

The event rate in cohort studies is typically calculated as the number of events per 100 person-years of follow-up, which accounts for variable follow-up times in a large-scale cohort. Since the same people will have had two or more tests for TB infection, the differences in event rates can be directly calculated, either as a risk difference or as a risk ratio. The incidence rate ratio can be estimated as incidence rate among people who test positive/incidence rate among people who test negative.

### Sensitivity and specificity

Current tests for TB infection generally have suboptimal sensitivity. Sensitivity is lower in children, PLHIV and other immunocompromised people. In these populations, superiority of a new test would be preferable to a noninferiority design.

Current IGRA tests have excellent specificity. Therefore, superior specificity is neither necessary nor likely to be demonstrable. Hence, a noninferiority design is sufficient for specificity when the reference standard test is an IGRA, or a TST in a population that has not been BCG-vaccinated. If the reference standard is TST in a BCG-vaccinated population, then a superiority design is recommended.

Sensitivity and specificity can be calculated using the standard formula based on the aforementioned reference standards. However, in a population with very low TB prevalence, specificity can be approximated using the total number of people tested as the denominator instead of the number of people without TB infection, as follows: all people who test negative/all people tested in the very-low-prevalence population.

### Concordance

If the new test is anticipated to have similar diagnostic accuracy to the reference test, but has operational advantages, such as lower cost or greater feasibility, then tests showing high agreement are valuable. The κ-statistics should be calculated, with the accompanying 95% confidence interval. κ-statistics account for chance-corrected agreement, which is especially important when prevalence of positive tests is either very low or very high. The sample size is determined by the maximum acceptable width of the κ 95% confidence interval, the underlying true proportion of positives and the anticipated value of κ [26].

## Technical issues

Immunoassays are complex assays influenced by multiple sources of variability which can impact results. Table 2 lists a range of typical sources of variability in *in vitro* tests for TB infection such as IGRA, which should be prioritised by developers in the technical description of the assay. A full list is available elsewhere [16].

**TABLE 2:** Typical sources of variability in interferon-γ release assay-like tests items for documentation

**Factors impacting stimulation assay**	Blood collection tubes: within- and between-lot variability
	Delay in blood processing and incubation time
	Volume of blood
**Analytical range of readout assay**	Limit of detection
	Lower limit of quantification
**Imprecision of the readout assay**	Intra-assay and inter-assay imprecision, in particular around the cut-off for test positivity
**Accuracy of readout assay**	Recovery
**Analytical specificity of readout assay**	Cross-reactivity
	Parallelism/dilution linearity
	Common interferents (*e.g.* rheumatoid factor, lipids, bilirubin, complement, haemolysate)
	Evaluation of curve-fitting model (≥5 determinations over multiple runs)
**Additional assessments**	Inter-laboratory imprecision (reproducibility)
	Analyte stability (freeze–thaw stability, short-term bench stability, long-term storage stability)

LoD: limit of detection; LLoQ: lower limit of quantification.

As immunoassays detect responses on a continuous scale, which is converted to a binary outcome as positive or negative by use of a threshold value (cut-off), a description of the variability around this cut-off is of particular relevance. To determine the degree of variability around the cut-off threshold of any new test, we recommend to evaluate changes in IGRA results by using an adequate number of participants without active TB with positive and negative IGRA values representative of normal physiological ranges in cohorts where prior infection is likely and reinfection events are rare. For example, studies in recent adult migrants from high TB incidence countries to low-to-middle incidence countries where TPT is not routinely initiated for a positive IGRA result could examine reproducibility of the initial IGRA positivity with minimal influence from reinfection. Similarly, reproducibility of IGRA results has often been evaluated in healthcare workers, demonstrating fluctuation of results [27]. Contacts of people with TB who recently converted to a positive test could also be followed-up to assess possibility of reversion. Samples should be used to estimate the rate of IGRA conversions/reversions using the predefined cut-off for assay positivity. In cases of IGRA conversion, a third sample may be collected to evaluate if conversion using the pre-defined cut-off was sustained due to an *M. tuberculosis* infection event, rather than a spurious effect of variability around the cut-off. Samples from such participants should be excluded when defining the range of the zone of uncertainty.

The QuantiFERON-TB Gold In-Tube and QuantiFERON-TB Gold Plus assays report considerable variability around the assay cut-off of 0.35 IU·mL^−1^ [28]. While the cut-off value for any new IGRA should be defined against the concordance with existing tests such as QuantiFERON-TB Gold Plus, manufacturers should be cautious about reporting test results around this zone of uncertainty. Assessment of any new IGRA should also report IU·mL^−1^ for the mitogen, the antigen and the unstimulated control separately. Each new IGRA will need to study zone of uncertainty. Assays with only a binary readout for TB infection should provide additional data to confirm reproducibility without resulting in a high number of invalid results. Participant samples from both populations with very low likelihood of TB infection as well as people with confirmed TB disease should be used to establish a range of interferon-γ responses expected for clinically relevant specimens.

## Evaluation of safety for skin tests

Safety of new skin tests should be evaluated against a reference skin test (TST) in a population representative for the target population for the new test to show that injection site reactions and other adverse events are similar to or fewer than with TST. Safety should be evaluated in various groups such as PLHIV, children and pregnant and lactating women. TST is safe to administer to pregnant women and lactating women. Similarly, it is unlikely that new skin tests for TB infection cause adverse effects on the fetus or nursing infants when administered to pregnant or lactating women.

The study design should seek to minimise bias in ascertaining local adverse reactions. One method is to give the new and the reference test at the same time in each forearm in a double-blinded manner. Another method to assess safety would be to compare adverse events reporting in a randomised controlled trial. However, trials are unlikely to be sufficient to detect rare adverse events and post-marketing surveillance is essential. Methods for recording adverse events should be adequate and clearly recorded, *e.g.* using MedDRA classification (www.meddra.org/). As skin tests are designed to induce a local reaction, it is important to pre-define how to interpret reactions as relevant indurations or adverse events.

## Economic evaluation

New tests should ideally have lower health system and patient costs compared to existing tests. In order to evaluate this aspect, costs associated with both the start-up and routine operations of the new test should be considered in studies that are evaluating the new test. Ideally, an economic evaluation should be incorporated into studies run at demonstration sites or independent research studies. Regardless of the study setting, it is important that an effort is made to document the “true” cost of implementing the new test (*i.e.* as it would be used in practice). The following costs should be considered: laboratory equipment and start-up; computers and software; supplies; cold-chain requirements; personnel time for different aspects of testing; initial and ongoing training; quality control and supervision; and health facility visits by patients with positive or negative tests. Optimum care organisation models should be used when implementation is being considered (*e.g.* “one-stop shop” where follow-up care for those with positive tests is coordinated and provided same day). Additional details on specific costs to consider during evaluations are available presented in the supplementary table.

## Operational characteristics

New tests for TB infection should also address operational challenges associated with the existing tests. The ability to deploy at the lowest level of the healthcare system is especially important. Instrument-free tests or tests that can be performed with a small, portable or hand-held instrument that function by battery or solar power are needed. Rapid tests (*e.g.* <1 h for results) would also offer a great advantage. For both skin tests and *in vitro* tests, the stability of reagents should be established under different conditions in accordance with the WHO standards for pre-qualification [29]. It is desirable that reagents are stable at high temperature and humidity for sufficient periods and that a cold chain is not required for their transportation. Tests that do not require the withdrawal of venous blood samples would be preferred.

## Concluding remarks

The uptake of TPT has been very slow globally, partly due to concerns about performance and operational challenges associated with current tests. New tests for TB infection offer an opportunity to facilitate the scale-up and targeting of TPT for more effective use. They also support other initiatives to improve TB preventive treatment, such as the new target product profiles released in 2020. Implementation of this framework for the evaluation of new tests for TB infection will facilitate standardised evaluation of new tests and will expedite their adoption into national policies at the scale needed to achieve global targets.

## Supplementary material

10.1183/13993003.04078-2020.Supp1 **Please note:** supplementary material is not edited by the Editorial Office, and is uploaded as it has been supplied by the author.
Supplementary table ERJ-04078-2020.Supplement

## Shareable PDF

10.1183/13993003.04078-2020.Shareable1 This one-page PDF can be shared freely online.
Shareable PDF ERJ-04078-2020.Shareable
