# Supplementary material for: Framework for the evaluation of new tests for tuberculosis infection
Source: Eur Respir J. 2021 Aug 19;58(2):2004078. doi: 10.1183/13993003.04078-2020 (PMC8374690; doi:10.1183/13993003.04078-2020)
Supplement: Supplementary file 1 [file ERJ-04078-2020.Supplement.pdf]

Supplementary table. Costs to be considered in economic evaluation of new tuberculosis infection tests

**Type of cost**	**Additional questions and information**
**Start-up costs**	
Laboratory equipment	If equipment will be used exclusively for testing for TB infection, include total cost of equipment<br>If equipment will not be used exclusively for testing for TB infection, specify approximate proportion of time that it will be dedicated to new test<br>For each piece of equipment, specify how long it is expected to last in order to depreciate capital costs accordingly
Laboratory space	Is additional space required for the new test for TB infection?
Initial calibration of equipment	Specify time requirements and job titles of people involved in task; or give cost of service contract
Licensing	Specify total cost for purchase of licence
Computers	Specify total cost for purchase of all new computers required<br>For each piece of equipment, specify how long it is expected to last in order to depreciate capital costs accordingly
Software	Specify total cost for purchase of all software required
Additional equipment (fridge, air-conditioner, generator)	Specify total cost for purchase of all new equipment<br>For each piece of equipment, specify how long it is expected to last in order to depreciate capital costs accordingly
Initial training	Specify total cost; or number, time requirements and job titles of people who would attend training
**Recurring costs**	
Ongoing calibration	Specify cost per session and frequency
Ongoing licensing	Specify renewal cost and frequency
Ongoing training	Specify total cost; or number, time requirements and job titles of people who would attend training
Equipment maintenance (per year)	Specify time requirements and job titles of people involved in task; or give cost of service contract
Quality assurance	Specify time requirements and job titles of people involved in task; or give cost of service contract<br>Specify supplies required to conduct quality assurance

Supplies required to administer test (e.g. gloves, syringes)	Ideally specify per-sample cost; or ensure units are otherwise clearly specified
Laboratory supplies for analysis	Ideally specify per-sample cost; or ensure units are otherwise clearly specified
Costs associated with cold chain	If cold chain is required, specify required items and their costs and units
Costs associated with shipping of samples	If samples are shipped to a laboratory, specify shipping costs per sample; specify per-sample costs, or ensure units are otherwise clearly specified
**Personnel**	
Approximate amount of time required to take sample	Specify time in minutes
Category of personnel who can obtain sample	Specify nurse or other personnel (provide details)
Approximate amount of personnel time required to process and analyse sample	Specify time in minutes
Category of personnel who can process or analyse sample	Specify laboratory technician or other personnel (provide details)
Approximate amount of time to interpret result	Specify time in minutes
Category of personnel who can interpret result	Specify laboratory technician, medical doctor or clerical staff

Note: For the reference standard, start-up costs should be estimated based on the current cost to repurchase equipment, licensing agreements, and so on.
