# Supplementary material for: Framework for the evaluation of new tests for tuberculosis infection
Source: Eur Respir J. 2021 Aug 19;58(2):2004078. doi: 10.1183/13993003.04078-2020 (PMC8374690; doi:10.1183/13993003.04078-2020)
Supplement: Supplementary file 2 [file ERJ-04078-2020.Shareable.pdf]

Framework for the evaluation of new tests for tuberculosis infection

SHAREABLE PDF

Yohhei Hamada[1,2], Saskia den Boon[3], Daniela Maria Cirillo[4], Adam Penn-Nicholson[5], Morten Ruhwald[5], Dick Menzies[6], Olivia Oxlade[6], Dennis Falzon[3], Avinash Kanchar[3], Alexei Korobitsyn[3], Matteo Zignol[3], Alberto Matteelli[7] and Tereza Kasaeva[3]

[1]Research Institute of Tuberculosis, Japan Anti-Tuberculosis Association, Tokyo, Japan. [2]University College London, London, UK. [3]Global TB Programme, World Health Organization, Geneva, Switzerland. [4]IRCCS San Raffaele Scientific Institute, Milan, Italy. [5]Foundation for Innovative New Diagnostics (FIND), Geneva, Switzerland. [6]McGill International TB Centre, Montreal, QC, Canada. [7]Collaborating Centre for TB/HIV Co-infection and TB Elimination, Dept of Infectious and Tropical Diseases, University of Brescia, Brescia, Italy.

Corresponding author: Yohhei Hamada (y.hamada@ucl.ac.uk)

Check for updates

@ERSpublications
The evaluation of new, improved tests for tuberculosis infection should be expedited using standard study protocols to accelerate adoption into policy and subsequent scale-up. A framework for such evaluation is described. https://bit.ly/38ChJbe

Cite this article as: Hamada Y, den Boon S, Cirillo DM, *et al*. Framework for the evaluation of new tests for tuberculosis infection. *Eur Respir J* 2021; 58: 2004078 [DOI: 10.1183/13993003.04078-2020].

This single-page version can be shared freely online.

Copyright ©The authors 2021

This version is distributed under the terms of the Creative Commons Attribution Non-Commercial Licence 4.0.

This article has supplementary material available from erj.ersjournals.com

Received: 4 Nov 2020
Accepted: 2 Jan 2021

Abstract

The scale-up of tuberculosis (TB) preventive treatment (TPT) must be accelerated to achieve the targets set by the United Nations High-level Meeting on TB and the End TB Strategy. The scale-up of effective TPT is hampered by concerns about operational challenges to implement the existing tests for TB infection. New simpler tests could facilitate the scale-up of testing for TB infection. We present a framework for evaluation of new immunodiagnostic tests for the detection of TB infection, with an aim to facilitate their standardised evaluation and accelerate adoption into global and national policies and subsequent scale-up. The framework describes the principles to be considered when evaluating new tests for TB infection and provides guidance to manufacturers, researchers, regulators and other users on study designs, populations, reference standards, sample size calculation and data analysis and it is also aligned with the Global Strategy for TB Research and Innovation adopted by the World Health Assembly in 2020. In addition, we briefly describe technical issues that should be considered when evaluating new tests, including the safety for skin tests, costs incurred by patients and the health system, and operational characteristics.
